# Effects of Different Management Practices on the Increase in Phytolith-Occluded Carbon in Moso Bamboo Forests

**DOI:** 10.3389/fpls.2020.591852

**Published:** 2020-12-03

**Authors:** Wanjie Lv, Guomo Zhou, Guangsheng Chen, Yufeng Zhou, Zhipeng Ge, Zhengwen Niu, Lin Xu, Yongjun Shi

**Affiliations:** ^1^State Key Laboratory of Subtropical Silviculture, Zhejiang A&F University, Hangzhou, China; ^2^Zhejiang Provincial Collaborative Innovation Center for Bamboo Resources and High-Efficiency Utilization, Zhejiang A&F University, Hangzhou, China; ^3^Key Laboratory of Carbon Cycling in Forest Ecosystems and Carbon Sequestration of Zhejiang Province, Zhejiang A&F University, Hangzhou, China; ^4^School of Environmental and Resources Science, Zhejiang A&F University, Hangzhou, China

**Keywords:** carbon sequestration, management practices, Moso bamboo forests, phytolith, PhytOC

## Abstract

Phytolith-occluded carbon (PhytOC), a promising long-term biogeochemical carbon sequestration mode, plays a crucial role in the global carbon cycle and the regulation of atmospheric CO_2_. Previous studies mostly focused on the estimation of the content and storage of PhytOC, while it remains unclear about how the management practices affect the PhytOC content and whether it varies with stand age. Moso bamboo (*Phyllostachys heterocycla* var. *pubescens*) has a great potential in carbon sequestration and is rich in PhytOC. Here, we selected four management treatments, including control (CK), compound fertilization (CF), silicon (Si) fertilization (SiF) (monosilicic acid can form phytoliths through silicification), and cut to investigate the variation of phytoliths and PhytOC contents in soil, leaves, and litters, and their storage in Moso bamboo forests. In soil, the SiF fertilizer treatment significantly (*P* < 0.05) increased phytolith content, PhytOC content, and storage compared to CK, while there were no significant differences between the treatments of CF and cut. In leaf, compared with CK, phytolith content of the second-degree leaves under SiF and the first-degree leaves under cut treatment significantly increased, and the three treatments significantly increased PhytOC storage for leaves with three age classes. In litter, the phytolith and PhytOC contents under the three treatments were not significantly different from that under the CK treatment. The PhytOC storage increased by 19.33% under SiF treatment, but significantly decreased by 40.63% under the CF treatment. For the entire Moso bamboo forest ecosystems, PhytOC storage of all the three management treatments increased compared with CK, with the largest increase by 102% under the SiF treatment. The effects of management practices on the accumulation of PhytOC varied with age. Our study implied that Si fertilization has a greater potential to significantly promote the capacity of sequestration of carbon in Moso bamboo forests.

## Introduction

How to increase terrestrial ecosystem carbon sequestration and thus mitigate global warming has been a long-term hotspot research area (Heimann and Reichstein, 2008; Pan et al., 2011; Peters et al., 2012; Taylor et al., 2015). Terrestrial biogeochemical carbon sequestration is one of the most promising approaches for long-term atmospheric CO_2_ sequestration (Parr et al., 2010; Song et al., 2013a). Occlusion of carbon within phytoliths (PhytOC) has been recently shown to be the most promising biogeochemical carbon sequestration mechanism (Song et al., 2012; Li et al., 2013b; Song et al., 2013a).

Bioavailable silicon (Si) in soil solution is absorbed by plant roots in the form of monosilicic acid (H_4_SiO_4_) (Meunier et al., 1999) and is mainly deposited in plant tissues (e.g., cell walls, cell lumina, and intercellular spaces of the cortex) to form the phytoliths (Casey et al., 2003; Cornelis et al., 2011; Zhang et al., 2016). Phytoliths exist in many plant tissues. A small quantity of organic carbon ranging from 0.1 to 5.8% can be occluded in plant phytoliths (Wilding, 1967; Parr et al., 2010; Zuo and Lü, 2011; Pan et al., 2017). During the litter decomposition process, phytoliths are released into soil and transferred to the subsoil (Parr and Sullivan, 2005; Fishkis et al., 2009; Pan et al., 2017). PhytOC is highly resistant to decompose and may accumulate in the soil for several thousands of years, as it is protected by phytolith silica (Wilding, 1967; Parr and Sullivan, 2005; Zuo et al., 2014). PhytOC contributes to approximately 82% of the total soil organic carbon (SOC) pool in some sediments after 2,000 years of decomposition (Parr and Sullivan, 2005), implying a high potential of PhytOC in the long-time biogeochemical sequestration of atmospheric CO_2_.

Previous studies have indicated that the accumulation and distribution of soil PhytOC depend not only on phytolith input from plant’s litter but also on phytolith outputs such as phytolith stability, harvesting loss, and phytolith transport (Blecker et al., 2006; Fishkis et al., 2009; Song et al., 2013b). Parr and Sullivan (2005) estimated that the cumulative flux of PhytOC in the subtropical and tropical regions was 7.2–8.8 kg ha^–1^ year^–1^, accounting for 37% of the global average SOC accumulative rate. Huang et al. (2014) reported that PhytOC accumulation could be 79 kg ha^–1^ year^–1^ in Lei bamboo forest soil. Zuo et al. (2014) estimated that 5.35 × 10^6^ t year^–1^ of PhytOC was stored in the upper soil of the Loess Plateau in China. Zhang et al. (2016) reported that the storage of soil PhytOC in Chinese fir forest, chestnut forest, and bamboo forest ranged from 0.96–1.40, 2.44–2.90, and 3.27–4.55 t ha^–1^, respectively. Therefore, the accumulation of soil PhytOC plays an important role in sequestering atmospheric CO_2_.

Moso bamboo is the most widely planted bamboo species in the subtropical regions of Asia, Africa, and Latin America, with a global total area of 31.5 million ha, which accounts for about 0.8% of the world’s total forest area in 2010 (Food and Agriculture Organization [FAO], 2010). Moso bamboo forest area is 6.01 million ha in China, accounting for about 73.8% of the total bamboo forests (Xu, 2014). Moso bamboo is widely distributed in southern China and has a long cultivation and utilization history (Xu, 2017), a high economic value, and a high carbon sequestration capability (Zhou, 2006; Zhou et al., 2011; Li et al., 2015). A previous study has indicated that the global potential for bio-sequestration via PhytOC in bamboo or other similar grass crops is 1.5 × 10^9^ Mg CO_2_ year^–1^, equivalent to 11% of the current increase in atmospheric CO_2_ (Parr et al., 2010). The present annual PhytOC sink in China’s forests was 1.7 ± 0.4 Tg CO_2_ year^–1^, and bamboo forests contributed by 30% (Song et al., 2013a).

Moso bamboo forests have a unique growth pattern, in which shoots usually begin to emerge from the ground at the end of March and complete height and diameter growth within the following 2 months (Li et al., 1998; Zhou et al., 2010; Song et al., 2016b). After this period, the diameter of breast height (DBH) and the height of culms remain constant owing to the Moso bamboo’s scarce secondary cambium, but the biomass begins to accumulate (Zhou et al., 2010; Xiao, 2015). After the completion of height growth, the old leaves begin to grow rapidly and then fall in the next spring, and in the meanwhile, the new leaves quickly emerge (Song et al., 2017). The leaf growth cycles every 2 years; if the Moso bamboo age is located within the first cycle, it is called “the first-degree bamboo” and within the second cycle, it is called “the second-degree bamboo,” and so on. Furthermore, as a result of long-term harvesting activities, the Moso bamboo forests are characterized by alternating off- and on-years (Chen, 1996; Zhou et al., 2010). In on-years, Moso bamboo forests have more shoot sprouts and slower rhizome root growth; in off-years, Moso bamboo forests have fewer shoot sprouts and faster rhizome root growth. This phenomenon usually alternates every 2 years. Moreover, the capacity of biomass accumulation of Moso bamboo tends to decrease rapidly after 4 years. Therefore, to maximize the economic benefits, farmers usually harvest Moso bamboo culms that are 4 years and older in November. Thus, Moso bamboo forests are uneven-aged forests with a 2-year interval (Zhou et al., 2010; Song et al., 2016a).

Previous studies only focused on the production of phytolith and PhytOC in bamboo forests. Few studies have investigated the effect of different management practices and the effects of age. Many previous studies have demonstrated that management practices could increase the aboveground biomass and SOC pool in the Moso bamboo forests (Zhou et al., 2006a,b; Qi et al., 2009; Li et al., 2017). Moreover, PhytOC sequestration is positively correlated with phytolith content, carbon content of phytolith, and the aboveground net primary production (ANPP) of plants. Therefore, we hypothesize that the treatment of management practices may increase PhytOC in Moso bamboo forests. Based on these hypotheses, our study objectives are (1) to analyze the PhytOC concentrations in Moso bamboo forests under different management practices, (2) to explore the effects of bamboo age on PhytOC concentrations whether these effects varied with age, and (3) to predict the PhytOC sequestration potential of Moso bamboo forests, under an optimized management practice.

## Materials and Methods

### Study Area and Experiment Sites

**FIGURE 1:**
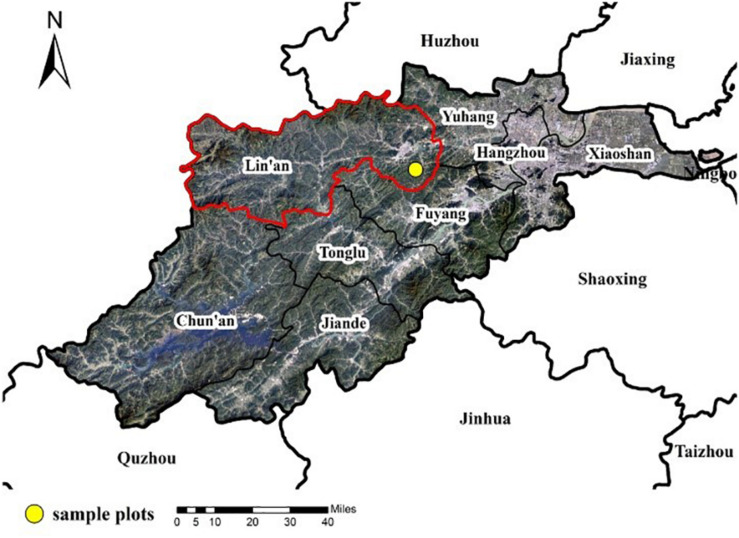
Location of the study region (red polygon) and the experimental site (yellow point) for Moso bamboo forests in Lin’an District, Hangzhou City, Zhejiang Province, China.

Our study was conducted in Lin’an District (119°45′E, 30°10′N), Hangzhou City, Zhejiang Province, in southeastern China (Figure 1). This region is characterized by a warm and humid subtropical monsoon climate. It has an average annual precipitation of 1,350–1,500 mm, most of which falls between May and August. The average annual temperature is 15.9°C with the highest temperature in July and the lowest in January. This area has undulating terrain, with elevation ranging from 90 to 200 m. As one of the first batch of the “Top Ten Bamboo Townships in China” and a key forest area in Zhejiang Province, Lin’an District is rich in bamboo resources. The city has 65,400 hectares of bamboo forests, and the annual output value of the bamboo industry is maintained at about 3 billion yuan (Fang, 2018).

The experimental sites were located at the southeastern Lin’an (Figure 1). The soils at the experimental sites are developed from siltstone and classified as a slightly acidic red soil in the Chinese system of soil classification (State Soil Survey Service of China, 1998), equivalent to the Ferralsols in the FAO soil classification system (FAO, 2006). The soil thickness is 50 cm. Before the treatments with management practices, the bamboo culms above 6 years old were harvested every 2 years since 2010. The culm density is 3,236 culms ha^–1^, with a mean DBH of 9.1 cm. The Moso bamboo forests have few understory shrubs and grasses. The sample plots were established in 2010, no tillage was applied, and the understory vegetation was retained.

### Experimental Design and Sampling Methods

The experiment was conducted from August 2016 to August 2019.

Four treatments, including compound fertilizer (CF), Si fertilizer (SiF), Cut (harvesting), and Control (CK), were deployed in the experimental sites in 2016. The detailed treatments are:

(i) Compound fertilizer (CF): Normal cut (removal of bamboo culms over 8 years old) and compound fertilizer application (1,800 kg ha^–1^, twice a year). The composition of compound fertilizer was N 13%, P 3%, K 2%, amino acid ≥ 8%, organic matter ≥ 15%, and humic acid ≥ 10%.
(ii) Si fertilizer (SiF): Normal cut (removal of bamboo culms over 8 years old) and Si fertilizer application (1,800 kg ha^–1^, twice a year). The composition of Si fertilizer was C concentration of 7 g kg^–1^ and SiO_2_ concentration of 723 g kg^–1^.
(iii) Cut: Intensive cut (complete removal of bamboo culms over 6 years old) and no fertilization.
(iv) Control groups (CK): normal cut and no fertilization.

A completely randomized block design with four management treatments and three replicates were deployed, and there were a total of 12 experimental plots (30 m × 30 m). To reduce the interference from the Moso bamboo rhizome spread to the adjacent plots, a 5-m-wide buffer zone was set up between every two experimental plots. The treatments were applied to the entire experimental plot, but the sample data were only collected from the center of each plot (20 m × 20 m).

Within each experimental plot, soil samples were taken from the 0–20 and 20–40-cm layers from five randomly selected points along two diagonal lines, and then mixed to form a composite sample for each layer. The samples were stored in clean plastic bags then brought back to the laboratory. Visible roots and plant fragments (>2 mm diameter) in soil were removed. The soil samples were then air dried at room temperature and ground to pass through a 2-mm sieve for chemical analysis. The soil samples for determining bulk density were collected using a bulk density corer (5.2 cm height and 3.5 cm radius) with a 100 cm^3^ volume. The wet soil weight was measured in each corer, and then the soils were dried at 105°C for 24 h to a constant weight. The water content (%) was then calculated.

The DBH of all bamboo culms with different ages in the 30 m × 30 m plots was measured, and the mean DBH in each age class was calculated. Ten bamboo culms close to the average DBH were selected to sample the bamboo leaves for each age class. The leaves were collected using a pruning shear, and the leaves from each age class were mixed. The leaf samples were brought back to the laboratory and rinsed with deionized water. The leaf samples were first dried at 105°C for half an hour and then further dried at 70°C to a constant weight and weighed. The leaf samples were ground as powders and passed through a 100-mesh sieve for chemical analysis.

We also collected all the dead branches and leaves (the new and partially decomposed litters) in three randomly selected 1 m × 1 m plots in each sample plot. The litter samples were stored in sealed plastic bags and weighed. The litter samples were brought back from the field and washed with deionized water, first dried at 105°C, further dried at 70°C to a constant weight, and then weighed. The dried samples were also ground to pass through a 100-mesh sieve for chemical analysis.

### Determination of Basic Soil Physicochemical Properties

In our study, the selected physicochemical properties of soil samples were measured using the following methods. Soil pH was determined using an acidity meter, namely, using a pH meter at a soil:water ratio of 1:2.5 (w/v). Soil moisture content was measured gravimetrically. The soil bulk density was determined using the equation below:

(1) $$\text{BD}=G*\frac{100}{\text{V}}*\left(100+\text{W}\right)$$

where BD is bulk density (g.cm^–3^), G is the wet soil weight in a corer (g), V is the volume of the corer (200 cm^3^), and W is the water content (%).

Alkali-hydrolyzed nitrogen was analyzed using the Alkali solution diffusion method. The soil available P concentration was measured using the Bary colorimetric method. The soil samples were first extracted with a mixed solution of NH_4_F (0.03 mol⋅L^–1^) and HCl (0.025 mol⋅L^–1^), and then the available P was measured colorimetrically using a spectrophotometer. The available K was determined using the ammonium acetate extraction flame photometer method. SOC’s content was oxidized by a certain amount of standard potassium dichromate solution and concentrated sulfuric acid under heating conditions, titrated by ferrous sulfate, and calculated by the consumed potassium dichromate (Bao, 2000).

(2) $${\text{C}}_{S\,O\,C}=\sum_{i=1,2}C_{i}\,D_{i}\,B_{i}\,100^{-1}$$

where C*_*SOC*_* is SOC reserves (mg kg^–1^), *i* is the soil profile layer (1: 0–20 and 2: 20–40 cm), C*_*i*_* is the SOC content of layer *i* (g kg^–1^), D*_*i*_* is the soil thickness of layer *i* (cm), and B*_*i*_* is the bulk density of layer *i* (g cm^–3^).

### Determination of Phytolith and PhytOC

Phytolith from leaves, litter, and soil was extracted by microwave digestion. Of soil, 0.40 g was digested with 4.8 mL of HNO_3_ and 4.8 mL of HCl in the digestion tube in the microwave digester. Then the liquid was transferred to a centrifugal tube for centrifugal cleaning. The extracted phytolith was placed in an oven, dried at 65°C for 48 h to a constant weight, then weighed (Parr, 2001; Parr et al., 2001).

The method of extracting phytolith from leaves and litter was slightly different from the above method. Soil samples (0.3 g), 5 mL of HNO_3_, 1 mL of H_2_O_2_, and 1 mL of HCl (added twice, 0.5 mL each time) were added into a digestion tube. The digestion tube was moved to a microwave digester for digestion; then the liquid was transferred to a centrifugal tube for centrifugal cleaning (Parr, 2001; Parr et al., 2001).

PhytOC was determined by alkali fusion spectrophotometry according to Yang et al. (2014). Put 0.01 g phytolith sample into a plastic centrifuge tube, add 0.5 mL of 10 mol⋅L^–1^ NaOH, shake well, and let it remain for 12 h. The solution was then transferred to a 30-mL glass centrifuge tube, 1 mL of 0.8 mol⋅L^–1^ K_2_Cr_2_O_7_ and 4.6 mL of H_2_SO_4_ were added, shaken gently, and placed in a 98° boiling water bath for 1 h. Finally, we cooled it and shook well at a constant volume, and took the supernatant for colorimetric determination after low-speed centrifugation. (Note: the weighing of the implant before and after the test used the same balance, which could not be calibrated in the middle).

In order to characterize the capacity of phytolith carbon sequestration, two parameters are selected: one is carbon content in phytolith and the other is PhytOC. Because different management practices can affect the phytolith content, then they will affect the carbon content in phytolith. In addition, PhytOC storage and SOC storage are two different ways of carbon sequestration, so it is meaningful to study the influence of different management practices on the sum of the two ways of carbon sequestration.

Soil phytolith concentration, C concentration in phytolith, PhytOC concentration, and soil PhytOC storage were calculated using the following equations:

(3) $$\mathrm{Soil}\,\mathrm{phytolith}\,\mathrm{concentration}\,\left(g\,{\mathrm{kg}}^{-1}\right)\;=\mathrm{phytolith}\,\mathrm{weight}\,\left(\text{g}\right)/\mathrm{soil}\,\mathrm{weight}\,\left(\text{kg}\right)$$

(4) $$C\,\mathrm{concentration}\,\mathrm{in}\,\mathrm{phytolith}\,\left(g\,{\mathrm{kg}}^{-1}\right)\;=C\,\mathrm{content}\,\mathrm{in}\,\mathrm{phytolith}\,\left(\text{g}\right)/\mathrm{phytolith}\,\mathrm{weight}\,\left(\text{kg}\right)$$

(5) $$\mathrm{Soil}\,\mathrm{PhytOC}\,\mathrm{concentration}\,\left(g\,{\mathrm{kg}}^{-1}\right)\;=C\,\mathrm{content}\,\mathrm{in}\,\mathrm{phytolith}\,\left(\text{g}\right)/\mathrm{soil}\,\mathrm{weight}\,\left(\text{kg}\right)$$

(6) $$\mathrm{Soil}\,\mathrm{PhytOC}\,\mathrm{storage}\,\left(\mathrm{kg}\,{\mathrm{ha}}^{-1}\right)\;=\sum_{i=1}^{n}B\,D_{i}\times t\,h_{i}\times s\,o\,i\,l\,P\,h\,y\,t\,O\,C\,c\,o\,n\,c\,e\,n\,t\,r\,a\,t\,i\,o\,n\,\left(g\,{\mathrm{kg}}^{-1}\right)\times 10000$$

where *i* is the soil profile layer (1: 0–20 and 2: 20–40 cm), *thi* is the thickness of each soil layer (cm), and *BDi* represents the soil bulk density for each layer (g cm^–3^).

Plant phytolith concentration, C concentration in phytolith, and PhytOC concentration were calculated the same as the soil. The PhytOC storage was calculated as follows:

(7) $$\mathrm{Plant}\,\mathrm{PhytOC}\,\mathrm{storage}\,\left({\mathrm{kgha}}^{-1}\right)\;=\mathrm{PhytOC}\,\mathrm{concentration}\,\left(g\,{\mathrm{kg}}^{-1}\right)\times \text{biomass}\,\left(\mathrm{kg}\,{\mathrm{ha}}^{-1}\right)\times 10^{-3}$$

### Statistical Analyses

The data presented in this paper were the average value of three replicates. Before performing the analysis of variance (ANOVA), the normality and homogeneity of variance were tested. ANOVA was conducted to examine factors and their interactive effects on the SOC concentration and storage, phytolith concentration, C concentration in phytolith (density of carbon contained in phytoliths), and the PhytOC concentration (density of carbon contained in the soil samples) and storage in Moso bamboo forest soils. When the ANOVA analysis indicated a significant treatment effect, the least significant difference (LSD) test was utilized to separate the means. An alpha level of 0.05 for significance was used in all statistical analyses, unless mentioned otherwise. The linear relationship between phytolith concentration and PhytOC storage in soil was determined. All statistical analyses were performed using R statistical software (R v3.2.1) (R Core Team, 2015).

## Results

### Soil Physicochemical Properties

**FIGURE 2:**
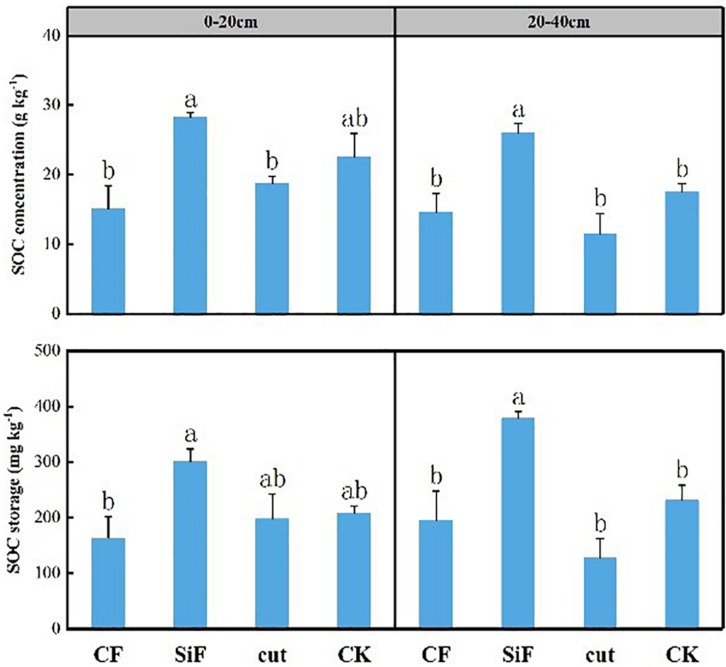
Concentration and storage of soil organic carbon (SOC) under four management practices in Moso bamboo forests. Different letters indicate significant (*p* < 0.05; *n* = 3) differences between treatments based on least significant difference (LSD) test. CF, compound fertilizer treatment; SiF, silicon fertilizer treatment; CK, control.

Si fertilization and cut practices significantly affected bulk density in the 20- to 40-cm soil layer (Table 1). SOC concentration under the treatments of CF, SiF, Cut, and CK were 14.67, 26.0, 11.47, and 17.6 mg kg^–1^, respectively. Compared with CK, SOC increased under SiF but decreased under CF and Cut treatments. The SOC concentration and storage under SiF significantly increased by 32.3 and 38.9%, respectively, compared with CK (Figure 2).

**TABLE 1:** Characteristics of soil physicochemical properties (means ± standard deviations) under different management treatments in Moso bamboo forests.

Soil depth	Management measures	pH	Bulk density (g cm^–3^)	Hydrolyzable N	Available P	Available K
0–20 cm	CF	4.93 ± 0.17a	1.077 ± 0.049a	79.33 ± 16.17a	18.30 ± 16.74a	3.73 ± 0.60a
	SiF	5.35 ± 0.66a	1.063 ± 0.097a	105.00 ± 12.12a	4.30 ± 3.00a	3.90 ± 0.89a
	Cut	4.85 ± 0.36a	1.043 ± 0.358a	86.33 ± 16.17a	13.38 ± 9.01a	3.70 ± 0.36a
	CK	5.52 ± 0.43a	0.957 ± 0.208a	86.33 ± 17.62a	14.51 ± 6.90a	3.97 ± 1.12a
20–40 cm	CF	4.91 ± 0.39a	1.307 ± 0.248ab	58.33 ± 16.17a	11.87 ± 2.62a	2.90 ± 0.46a
	SiF	5.60 ± 0.77a	1.463 ± 0.083a	56.00 ± 21.00a	7.70 ± 3.00a	2.73 ± 0.59a
	Cut	4.87 ± 0.38a	1.093 ± 0.110b	51.33 ± 14.57a	10.35 ± 4.59a	3.03 ± 0.61a
	CK	5.01 ± 0.11a	1.310 ± 0.232ab	56.00 ± 7.00a	8.46 ± 2.36a	2.87 ± 0.64a

*Note. Different letters indicate significant (*p* < 0.05; *n* = 3) differences between treatments based on least significant difference (LSD) test. CF, compound fertilizer treatment; SiF, silicon fertilizer treatment; CK, control.*

### Phytolith Content Under Different Management Practices

**FIGURE 3:**
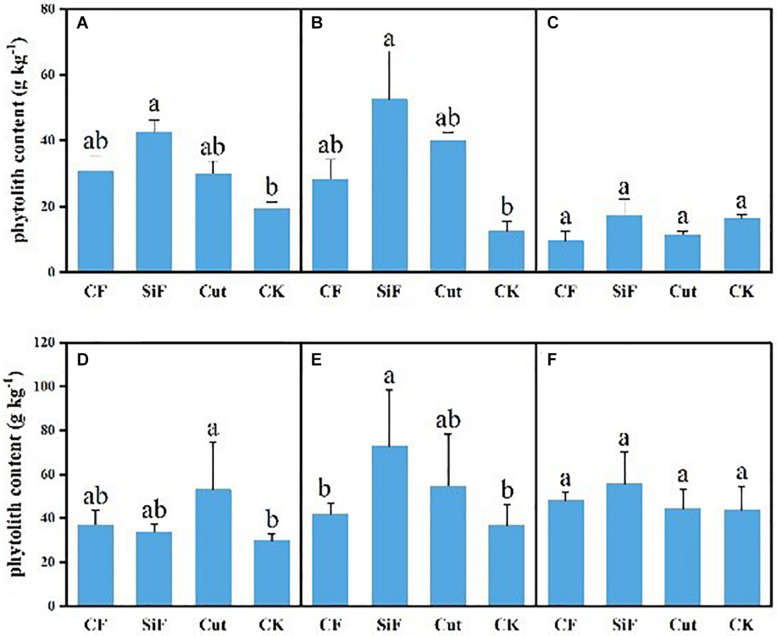
Phytolith content (g kg^–1^) under different management treatments for different forest components. **(A)** 0–20-cm soil layer. **(B)** 20–40-cm soil layer. **(C)** Litter. **(D)** First-degree leaves. **(E)** Second-degree leaves. **(F)** Third-degree leaves. Error bars represent the standard deviations of the means. Different lowercase letters indicate significant differences among treatments at a significance level of *p* < 0.05 based on the LSD statistic test. CF, compound fertilizer treatment; SiF, silicon fertilizer treatment; CK, control.

For CF and cut treatments, phytolith content had no significant difference with that under CK treatment for both soil layers, while SiF significantly increased phytolith content by 54.8% in the topsoil (0–20 cm) and 76.2% in the subsoil layer (20–40 cm) (Figure 3). The phytolith content of leaves under four management treatments varied from 29.73 to 72.97 g kg^–1^. For the first-degree leaves, the phytolith content under the cut treatment was significantly higher (78.36%) than that under CK, while there were no significant differences among three treatments. For the second-degree leaves, the phytolith content under SiF treatment was significantly higher than that under CK and CF treatment, while there were no significant differences with the cut treatment. For the third-degree leaves, the phytolith content was not significantly different among the four treatments. For litters, although the phytolith content was higher under the three treatments, the difference was not significant compared with the CK (Figure 3C).

### Content and Storage of PhytOC Under Different Management Practices

**FIGURE 4:**
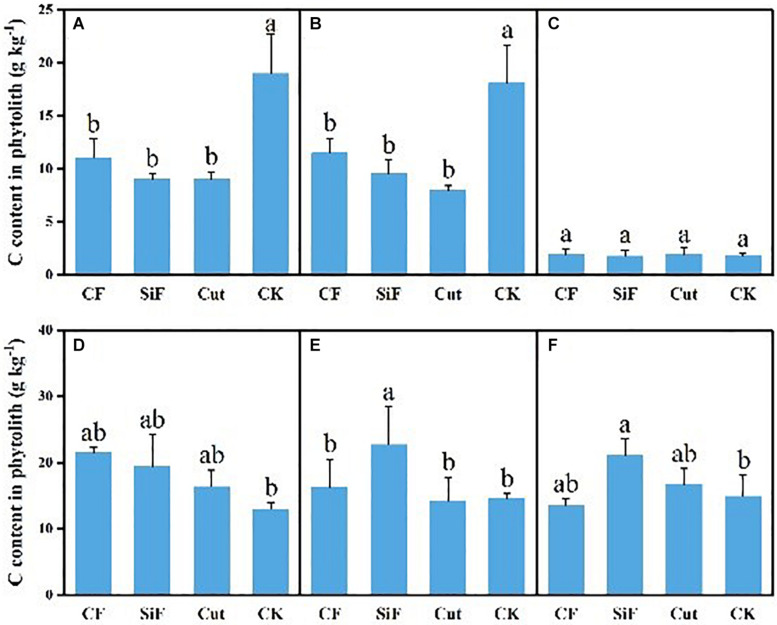
Carbon content in phytolith (g kg^–1^) under different management treatments for different forest components. **(A)** 0–20-cm soil layer. **(B)** 20–40-cm soil layer. **(C)** Litters. **(D)** First-degree leaves. **(E)** Second-degree leaves. **(F)** Third-degree leaves. Error bars represent the standard deviations of the means. Different lowercase letters indicate significant differences among treatments at a significance level of *p* < 0.05 based on the LSD statistic test. CF, compound fertilizer treatment; SiF, silicon fertilizer treatment; CK, control.

**FIGURE 5:**
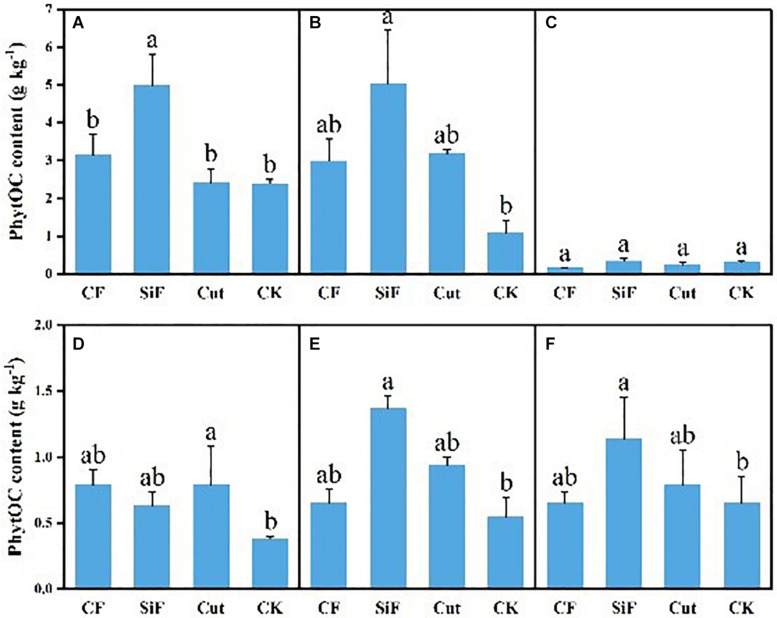
PhytOC content (g kg^–1^) under different management treatments for different forest components. **(A)** 0–20-cm soil layer. **(B)** 20–40-cm soil layer. **(C)** Litterfall. **(D)** First-degree leaves. **(E)** Second-degree leaves. **(F)** Third-degree leaves. Error bars represent the standard deviations of the means. Different lowercase letters indicate significant differences among treatments at a significance level of *p* < 0.05 based on the LSD statistic test. CF, compound fertilizer treatment; SiF, silicon fertilizer treatment; CK, control.

**FIGURE 6:**
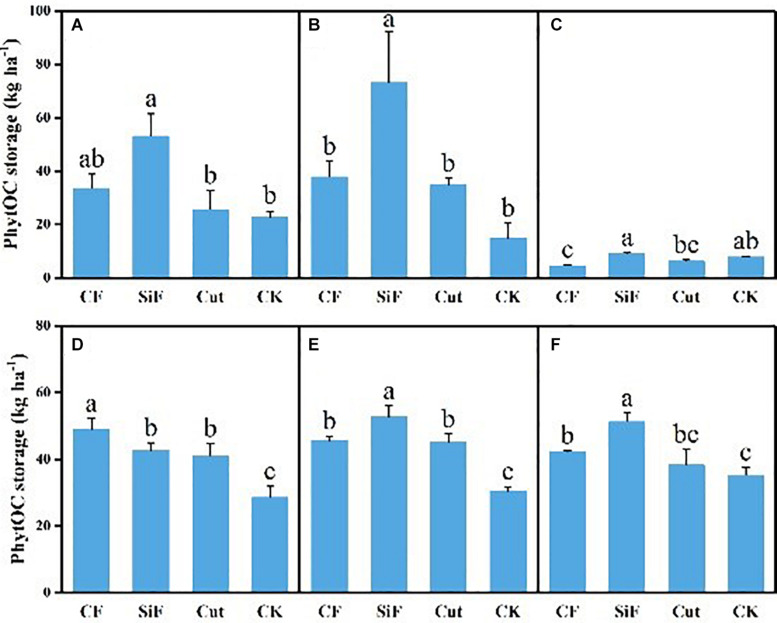
PhytOC storage (kg ha^–1^) under different management treatments for different forest components. **(A)** 0–20-cm soil layer. **(B)** 20–40-cm soil layer. **(C)** Litter. **(D)** First-degree leaves. **(E)** Second-degree leaves. **(F)** Third-degree leaves. Error bars represent the standard deviations of the means. Different lowercase letters indicate significant differences among treatments at a significance level of *p* < 0.05 based on the LSD statistic test. CF, compound fertilizer treatment; SiF, silicon fertilizer treatment; CK, control.

A startling contrast was found in the carbon content of phytolith in the soil. The result demonstrated decreased content while showing little difference among the three groups except CK. The carbon content of phytolith in both soil layers was significantly reduced under the three treatments compared with CK, while it was not significant among the three treatments (Figure 4A). The SiF treatment significantly increased PhytOC content by 52.2% in the 0–20-cm layer and 78.6% in the 20–40-cm layer, respectively, compared with CK, while there was no significant difference among CF, Cut, and CK (Figure 5). In the entire 0–40-cm soil layer, the PhytOC storage ranged from 14.8 to 73.13 kg ha^–1^ under four management practices and significantly increased under the application of the SiF treatment by 133% in the topsoil layer and 394% in the subsoil layer compared with CK, respectively (Figure 6). The carbon content of soil phytolith in the soil decreased under three treatments compared with CK, while the PhytOC storage increased significantly, implying that there was a significant negative correlation between the two indexes in the soil between these two variables.

For the first-degree leaves, the carbon content in phytolith was not significantly different among four treatments. For the second-degree leaves, SiF treatment significantly increased the carbon content in phytolith and no significant differences under the other three treatments. For the third-degree leaves, SiF treatment also significantly increased the carbon content in phytolith compared with CK, but not significantly different from that under CF and Cut treatments (Figure 4). The SiF treatment significantly increased the PhytOC content in all leaf age classes, while no significant differences exist among the other three treatments (Figure 5). Compared with CK, the three treatments significantly increased the PhytOC storage for all classes of leaves. For the mature age classes (second and third degree), the effects of SiF treatment on the PhytOC storage were the largest, increasing by 72.5% for the second-degree leaves and 45.4% for the third-degree leaves, respectively, compared with CK. In contrast, the effects of SiF reduced when the leaves were young (first degree), and CF had the largest effects on PhytOC storage, increased by 71.43% (Figure 6).

For the litters, the effects of treatments on PhytOC and phytolith carbon contents were similar (Figures 4, 5). The CF and Cut treatments decreased the PhytOC content by 48.72 and 23.64%, respectively, compared with CK, while the SiF treatment increased PhytOC content by 10.51% (Figure 5). The effects of treatments on PhytOC storage and PhytOC content were consistent, with an increase of 19.33% under SiF treatment, and a decrease of 40.63 and 20.25% under CF and Cut treatments, respectively (Figure 6).

Different from the soil, there was a positive correlation between the phytolith content and the organic carbon content in phytoliths of litter and leaf.

**FIGURE 7:**
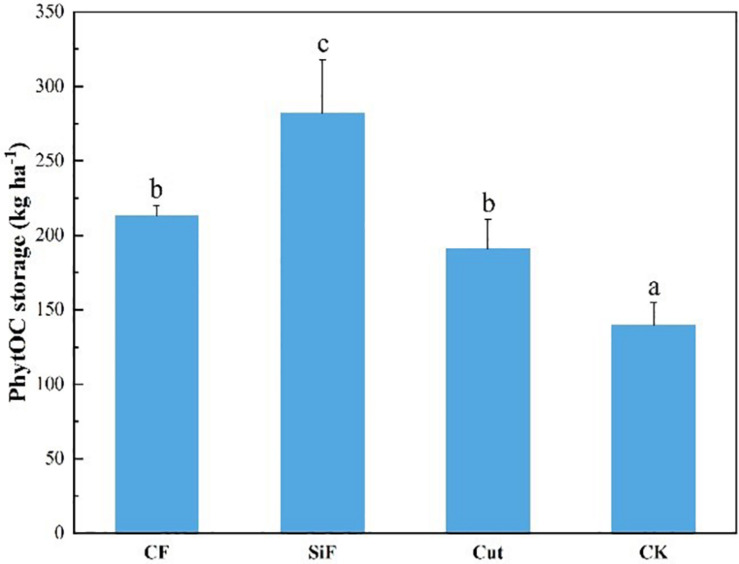
PhytOC storage of the entire Moso bamboo forest ecosystem (soil + litter + leaves) under four management treatments. CF, compound fertilizer treatment; SiF, silicon fertilizer treatment; CK, control. Different lowercase letters indicate significant differences among treatments at a significance level of *p* < 0.05 based on the LSD statistic test.

Compared with CK, the other three management treatments can significantly increase the PhytOC storage in the Moso bamboo forest ecosystems (Figure 7). However, no significant difference was found between the treatments of CF and Cut. Among the four management treatments, the increase in PhytOC storage during the study period was the largest under the treatment of SiF (102%), followed by CF (34.4%) and cut treatments (26.9%).

## Discussion

### Moso Bamboo Forest SOC Sink Under Different Managements

The analysis of SOC storage showed that the application of SiF could improve soil fertility and thus significantly increase SOC storage in Moso bamboo forests. The result coincided with a previous study (Xu, 2019). In Xu’s research, compared with CK, the SOC storage increased by 45.52 CO_2_-eq Mg ha^–1^, high-efficiency water-soluble Si fertilizer increased SOC storage by 51.94 CO_2_-eq Mg ha^–1^ under Si1 (0.225 t ha^–1^) treatment and 53.68 CO_2_-eq Mg ha^–1^ under Si3 (1.125 t ha^–1^) treatment. Song et al. (2018) studied the SOC stabilization by regulating Si and found that positive relationships existed between biogenic Si and SOC. We demonstrated that the application of SiF increased the SOC of Moso bamboo, shrubs, and herbs on the sample plots. After the litters of these bamboo leaves, shrubs, and herbs decomposed and transferred into soil, the SOC at the top soil layer would also increase. Li et al. (2017) found that fertilization and cut can significantly affect SOC in the Moso bamboo forests. They found that both large-scale fertilization with intensity cut and large-scale fertilization with moderate cut can cause a decrease in SOC storage, while both the large-scale fertilization with weak cut and medium-scale fertilization with weak cut can increase SOC storage. Their results indicated that the SOC storage of bamboo forests decreased significantly after intensive management, while the reasonable combination of management practices could significantly increase the SOC storage and improve the carbon sink capacity. Our study showed similar results that the management practices of CF and cut reduced the SOC storage, but with no statistically significant difference (*p* > 0.05). Du (2013) studied the effect of different fertilizer applications on the carbon storage of bamboo forest ecosystems and found that compared with no fertilization, the SOC under all fertilization treatments decreased in varying degrees. In addition, the effect of bamboo special fertilization on SOC was greater than that of organic fertilizer and NPK formula fertilizer. The reason may be that the large-scale application of CF can provide additional organic matter and nutrients to the forests, and accelerate the decomposition and transformation of organic matter. In addition, human activities such as harvesting (cut) down and bamboo shoots digging, and understory vegetation controls can result in nutrient losses from Moso bamboo forests, which further reduced the litter mass and soil organic matter accumulation.

### Effects of Different Management Measures on the Accumulation of Phytoliths

In this study, phytolith contents in both soil and leaves increased under all the three management treatments compared with CK, and significant differences were found under SiF treatment. SiF can significantly increase the available Si content in soils and thus the plant organs of Moso bamboo forests, which can promote the formation of phytolith since Si is an important component in phytolith. This pattern is in accordance with a recent study (Li et al., 2020b) that has showed that the addition of SiF can increase available Si in soil.

We also found that the increase in phytolith content in soil was much greater in leaves and litter under the three treatments compared with the control. This can be explained by the transpiration of leaves that can drive the Si transport and deposit to the root, stem, leaf, and shoots of Moso bamboo in the form of monoclinic acid. Thus, the Si fertilization can significantly increase the Si contents in plant organs, and then through litterfall and its decomposition, the soil phytolith content could also increase. It is necessary to further explore the effects of Moso bamboo litter on the carbon sink of bamboo forest ecosystems to help clarify the relationship between carbon flow between soil and vegetation components, which provides an essential basis for understanding the carbon sink potential of bamboo forest ecosystems (Hu, 2019). Our result is also in good agreement with recent studies, indicating that SiF addition increased phytolith concentration (Si uptake) in wheat (Liu et al., 2014) and rice (Sun et al., 2019; Li et al., 2020a), as well as bamboo (Huang et al., 2020). Certainly, the different impact magnitude under Si fertilization may be caused by the variations in vegetation types, source of SiF, and soil Si availability (Li and Delvaux, 2019; Huang et al., 2020).

We also found that the effects of management practices on phytolith contents varied with age, i.e., the phytolith content increased from the third-degree leaves to the second-degree leaves, but decreased to the first-degree leaves in the Moso bamboo forests (Figure 5). This result could be due to the following reasons. First, the development and accumulation rate of dry matter of third-degree bamboo forests slowed down or kept no change, so there was no significant difference among the four management treatments. Second, the second-degree bamboo was in its fast growth stage, and its capability to absorb nutrients (e.g., Si) was stronger. Combined with the characteristics of SiF mentioned above, the SiF applied to the second-degree leaves can significantly increase phytolith content. Finally, for the first-degree bamboo, its capability to absorb nutrients such as Si was weak, so the SiF and CF treatments did not significantly change the phytolith contents compared with the control. However, when some Moso bamboo culms was harvested, the stand density reduced, the competition among bamboos weakened and thus increased the area directly irradiated by the sun and the absorption of CO_2_ per unit area of bamboo leaves, while the change in atmospheric CO_2_ concentration affects the formation and size of phytoliths by affecting the photosynthetic rate of plants (Ge et al., 2010). Therefore, with the photosynthetic capacity enhanced, the phytolith contents of first-degree leaves under the cut treatment significantly increased compared with the control.

Interestingly, compared to the control, our data further showed that the phytolith content in litter decreased under the treatments of CF and cut. In contrast, the CF and cut treatments significantly increased soil and leaf phytolith contents, and the SiF treatment increased soil, leaf, and litter phytolith content. This phenomenon is probably because after Moso bamboo leaves experienced the stress effect and decay, the treatment’s effect on phytolith content started to decrease, and the growth began to decline, even lower than that of the control. However, the growing base under SiF treatment was larger initially, so the final phytolith content was still increasing. Besides, cut affected the amount and decomposition rate of litter (Paudel, 2015). The removal of the accumulation from the forest will reduce the stand density, increase the area directly irradiated by the sun, and increase the ground temperature, which will accelerate the decomposition of litter and reduce the amount of litter (Hu et al., 2007). Li et al. (2018) studied the existing amount of litter in the semi-decomposed layer of natural spruce-fiber and broad-leaved mixed forests and found that the management effects can be ranked as severe logging > control > light logging > moderate logging. Ying (2015) also studied the litter carbon sinks and fluxes in key subtropical forest types in China and found that the content of phytoliths in Moso bamboo forests was significantly different from, and higher than, the other three forest types.

### Effects of Management Practices on Phytolith Carbon Sequestration

Among all the management treatments, our analysis suggested that carbon content in phytolith of the soil had decreased significantly by 41.8, 52.2, and 52.6% at the topsoil layer and 36.4, 47.1, and 55.9% at the subsoil layer under the treatments of CF, SiF, and cut, respectively, compared with the control. However, there was no significant difference among the three management treatments. This was opposite to the management effects on phytolith content. The change in PhytOC content was in line with phytolith content under the three management measures, i.e., soil PhytOC content showed a significant difference (*p* < 0.05) under the treatment of SiF, but no significant difference was found in the leaves and litters. This was probably because the application of SiF increased the absorption of soluble Si by higher plant roots, and then the effect was transported to other organs such as stems, leaves, and roots, through the transduction tissue, and finally precipitated in the plant cells and came down (Zhang and Li, 2020).

The application of SiF can significantly affect soil PhytOC storage. A recent study (Sun et al., 2019) found that no matter whether the paddy soil was lacking in Si or rich in Si, the application of SiF significantly improved the Si contents of rice organs, such as stem, sheath, leaf, grain, and root. This is consistent with our results. This finding is also in good agreement with Li et al. (2013a), who showed that regulating Si supply might increase plant PhytOC content. In this respect, the combination of Si supply may enhance the contents of bamboo phytolith and OC occluded within phytoliths in bamboo forests. Guo et al. (2015) applied basalt powder to paddy soil and found that Si-rich fertilizer could distinctly increase the phytolith and PhytOC content of rice tissues, and increase the phytolith carbon production flux 1.5 times in the meantime. The PhytOC storage in leaves presented significant differences of the mature leaves and had not reached a significant level of the young leaves, which indicated from the side that SiF has a stress effect on the Moso bamboo. We attributed this result to the fact that in the year when SiF was applied, the fertilizer has a stimulating effect on the bamboo leaves and increased the phytOC storage. As time passed, the effect on the phytolith of the bamboo forest leaves began to move down and transferred to the soil, and the leaves entered a stable stage, so it did not reach a significant level of young leaves. Although CF and cut did not reach a significant level, they could substantially improve the PhytOC storage through increasing phytolith accumulation. In the same way, the change in PhytOC storage is as consistent with the phytolith content in the litter. This indicated that the increased PhytOC storage was the result of the accumulation of phytolith content rather than increased carbon content in phytolith. Namely, increasing phytolith content can promote the potential of phytolith C sequestration. This result was similar to that of Huang et al. (2014) and Yang et al. (2018). In addition, the carbon content of phytolith in bamboo leaves and litter is not inversely proportional to the phytOC storage and may be related to other factors, such as the biomass in bamboo forests (Li et al., 2013a).

On the whole, both PhytOC storage and SOC storage show higher carbon storage under SiF, that is, the application of SiF had better capacity of carbon sequestration under these four management practices.

## Conclusion

In this study, we explored how the management practices affected phytolith content and PhytOC content and whether the effects varied with age of Moso bamboo forests. The results were summarized as follows: (i) In soil, compared with CK, phytolith content and PhytOC storage increased under the three management treatments, both in the topsoil and subsoil layer, and the treatment of SiF had achieved statistically significant increase. The management effects on overall PhytOC storage can be ranked as: SiF > CF > cut > CK. (ii) In leaf, compared with CK, the phytolith content had all increased under the three treatments, and the management effects on overall PhytOC storage varied as a following trend: SiF > CF > cut > CK. Importantly, the phytolith content in the leaves under different treatments varied with age. Compared with CK, the management treatments showed no significant impacts on the phytolith content in the third-degree leaves; the SiF treatment significantly increased phytolith content in the second-degree and the first-degree leaves. (iii) In litter, the management effects on PhytOC storage and phytolith content were similar. Both decreased under the treatment of CF and cut, but increased under the SiF treatment. The management impacts on overall PhytOC storage varied in the following order: SiF > CK > cut > CF. (iv) For the entire Moso bamboo forest ecosystems, PhytOC storage under the three management treatments increased significantly compared with CK, but there were no significant differences between CF and cut treatments. Our results suggested that Si fertilization is an effective way to promote the PhytOC sequestration in Moso bamboo forests via improving the phytolith accumulation.

## Data Availability Statement

The raw data supporting the conclusions of this article will be made available by the authors, without undue reservation.

## Author Contributions

All authors listed have made a substantial, direct and intellectual contribution to the work, and approved it for publication.

## Conflict of Interest

The authors declare that the research was conducted in the absence of any commercial or financial relationships that could be construed as a potential conflict of interest.
